# A review on the role of nanoparticles for targeted brain drug delivery: synthesis, characterization, and applications

**DOI:** 10.17179/excli2024-7163

**Published:** 2025-01-03

**Authors:** Payam Nawzad Mohammed, Narmin Hamaamin Hussen, Aso Hameed Hasan, Hozan Jaza Hama Salh, Joazaizulfazli Jamalis, Sumeer Ahmed, Ajmal R. Bhat, Mohammad Amjad Kamal

**Affiliations:** 1 Department of Pharmacognosy and Pharmaceutical Chemistry, College of Pharmacy, University of Sulaimani, Sulaimani 46001, Kurdistan Region-Iraq, Iraq; 2 Department of Chemistry, College of Science, University of Garmian, Kalar 46021, Kurdistan Region-Iraq, Iraq; 3 Department of Chemistry, Faculty of Science, Universiti Teknologi Malaysia- 81310 Johor Bahru, Johor, Malaysia; 4 Department of Clinical Pharmacy, College of Pharmacy, University of Sulaimani, Sulaimani 46001, Kurdistan Region, Iraq; 5 Post-Graduate and Research Department of Chemistry, The New College (Autonomous), University of Madras, Chennai - 600014, India; 6 Department of Chemistry, RTM Nagpur University, Nagpur- 440033, India; 7 Pharmaceutical Sciences, College of Pharmacy, Princess Nourah Bint Abdulrahman University, Riyadh, Saudi Arabia; 8 Department of Pharmacy, Faculty of Health and Life Sciences, Daffodil International University, Birulia, Savar, Dhaka -1216, Bangladesh; 9 Centre for Global Health Research, Saveetha Medical College and Hospital, Saveetha Institute of Medical and Technical Sciences, Chennai, Tamil Nadu, India; 10 Novel Global Community Educational Foundation, Australia

**Keywords:** nanoparticles, brain disorders, BBB, methods, synthesis, applications

## Abstract

Unfortunately, nowadays, brain disorders, which include both neurological and mental disorders, are the main cause of years spent living with a disability worldwide. There are serious diseases with a high prevalence and a high mortality rate. However, the outmoded technical infrastructure makes their treatment difficult. The blood-brain barrier (BBB) serves as a protective mechanism for the central nervous system (CNS) and regulates its homeostatic processes. The brain is protected against injury and illness by an extremely complex system that precisely regulates the flow of ions, very few tiny molecules, and an even smaller number of macromolecules from the blood to the brain. Nevertheless, the BBB also considerably inhibits the delivery of medications to the brain, making it impossible to treat a variety of neurological diseases. Several strategies are now being studied to enhance the transport of drugs over the BBB. According to this research, nanoparticles are one of the most promising agents for brain disease treatment while many conventional drugs are also capable of crossing this barrier but there are amazing facts about nanoparticles in brain drug delivery. For example, 1. Precision Targeting: Through mechanisms such as receptor-mediated transport, ligand attachment, or the use of external stimuli (e.g., magnetic or thermal guidance), nanoparticles can deliver drugs specifically to diseased areas of the brain while minimizing exposure to healthy tissues. This targeted approach reduces side effects and enhances therapeutic outcomes. 2. Improved Drug Stability: Drugs can be encapsulated by nanoparticles, which keeps them stable and shields them from deterioration while being transported to the brain. 3. Therapeutic Payload: Nanoparticles possess a high surface-area-to-volume ratio, enabling them to encapsulate a substantial quantity of therapeutic agents relative to their size. This allows for enhanced drug delivery efficiency, maximizing therapeutic outcomes while potentially reducing the required dosage to achieve the desired effect. 4. Imaging Properties: Certain nanoparticles can also act as contrast agents for magnetic resonance imaging (MRI), allowing for the real-time visualization of drug distribution and administration in the brain. 5. Combination Therapy Possibility: Nanoparticles can be designed to co-deliver multiple medications or therapeutic agents, which could enhance synergistic effects. There have been *in vivo* studies where nanoparticles were successfully used for combination therapies, demonstrating potential for personalized treatments. One notable example is in cancer treatment, where nanoparticles have been designed to co-deliver multiple chemotherapeutic agents. In general, brain medication delivery by nanoparticles is a novel strategy that has the potential to revolutionize neurological disease therapy and enhance patient outcomes. The study furthermore includes a concise depiction of the structural and physiological characteristics of the BBB, and it also provides an overview of the nanoparticles that are most often used in medicine. A brief overview of the structural and physiochemical characteristics of the NPs, as well as the most popular nanoparticles used in medicine, is also included in the review.

## List of Abbreviations

AD: Alzheimer's Disease

AMT: Adsorptive-mediated transcytosis

BBB: Blood-brain barrier

CNS: Central nervous system

CSF: Cerebrospinal fluid

CVD: Chemical vapor deposition 

DNA: Deoxyribonucleic acid

GQDs: Graphene quantum dots

INPs: Inorganic nanoparticles 

MS: Multiple sclerosis

NCs: Nanocarriers 

NMDA Receptor: *N*-methyl-D-aspartate receptor

NPs: Nanoparticles

ONPS: Organic nanoparticles 

PD: Parkinson's Disease

PEG: Polyethylene glycol

PEI: Polyethyleneimine

QDs: Quantum dots

RMT: Receptor-mediated transcytosis

ROS: Reactive oxygen species

SLN: Solid lipid nanoparticles

## Introduction

As molecular biologist Francis Crick says, "There is no scientific study more vital to man than the study of his own brain. Our entire view of the universe depends on it”. Because the brain is the control center of our body diseases that affect the brain are more terrifying than those affecting other organs of the human body (Hodler et al., 2020[35]). Any serious disease has the potential to impair our ability to do things and may cause pain, suffering, or even death (Liu et al., 2023[58]). But brain disorders can impact the entire body, including cognition, motor function, remembering, and managing our emotions, they even alter our personalities (Mistretta et al., 2023[66]). About 300 psychiatric conditions and more than 600 neurological diseases exist nowadays (Liu et al., 2023[58]; Pinheiro et al., 2021[78]). Brain disorders encompass a range of conditions such as brain injuries, brain tumors, neurodegenerative illnesses, mental disorders, stroke, neurodevelopmental disorders, and epilepsy. These conditions are significant contributors to mortality and disability in adults (Mistretta et al., 2023[66]; Sakai and Yamada, 2019[84]). Most of these disorders can put the human life in a danger; 12 % of total deaths are due to brain diseases around the world (Halamoda-Kenzaoui et al., 2019[31]). Alzheimer disease (AD) and other dementias are predicted to account for 2.84 % of all deaths from neurological diseases (Khan et al., 2018[49]), while cerebrovascular disease accounted for 8 % of all fatalities in high-income nations in 2005 (Bastogne, 2017[9]). Many of these disorders are incurable, and there has been no specific treatment until now (Bastogne, 2017[9]; Nikalje, 2015[71]). Despite the fact that many promising biopharmaceutical agents have been created, only a small percentage of them (less than 5 %) can be used to treat the central nervous system because the blood-brain barrier prevents them from reaching the desired site of action in therapeutically significant amounts (Nikalje, 2015[71]; Pinheiro et al., 2021[78]).

The majority of large-molecule therapeutic candidates for disorders of the central nervous system such as monoclonal antibodies, nucleic acids and genes, peptides, and recombinant proteins never reach the clinic for the same reason (Gao, 2016[29]). Researchers are searching for alternative targeted drug delivery approach to minimize the side effects of a therapeutic agent (Kisała et al., 2018[52]). Different strategies are used to develop, and in this respect, nanoparticles are the best option materials. Recently, medical researchers are paying more attention to nanoparticles (NPs), which are thought to be a viable tool for therapeutic development (Anwar, 2018[3]; Halamoda-Kenzaoui et al., 2019[31]; Hussen et al., 2024[38]). They have been cited as desirable candidates for developing medication penetration through BBB because they have special characteristics of nanometric size, surface charge and shape, these properties give the idea that nano-delivery devices are suitable for brain delivery particularly the interaction and recognition among and the molecules expressed in the desired region of the brain and ligands conjugated on the surface of the nanoparticle (Kisała et al., 2018[52]; Skotland et al., 2014[92]). This makes nanoparticles the key biological effects, safety, efficacy and enables them to bind with cells and trigger a cell reaction. Another point is that nanosystems are important to minimize the negative side effects and reduce toxicity (Thomsen et al., 2015[99]). Nanotechnology plays a vital role in developing advanced systems that enable precise delivery of therapeutic and diagnostic substances to specific areas of the brain. Nanotechnology reduces the random spread of medications, which in turn reduces negative side effects, increases the amount of drug at the target site, and ultimately improves the effectiveness of treatment. Within the complex structure of the central nervous system (CNS), the careful implementation of nanotechnological methods shows potential for overcoming the obstacles that hinder the successful treatment of neurological illnesses (Pinheiro et al., 2021[78]; Walter et al., 2021[103]; Wohlfart et al., 2012[108]).

The objective of this review is to provide an overview of research articles that have been published over the last 10 years on the special properties and high ability of nanoparticles to cross blood-brain barrier (BBB). To do this, it is necessary to first describe BBB, followed by a detailed description of the ways that NPs can cross BBB, the most efficient methods for synthesis of nanoparticles, and finally review the literature focusing on the usage of nanoparticle properties on neurodegenerative diseases.

## Blood-Brain Barrier (BBB)

**Figure 1:**
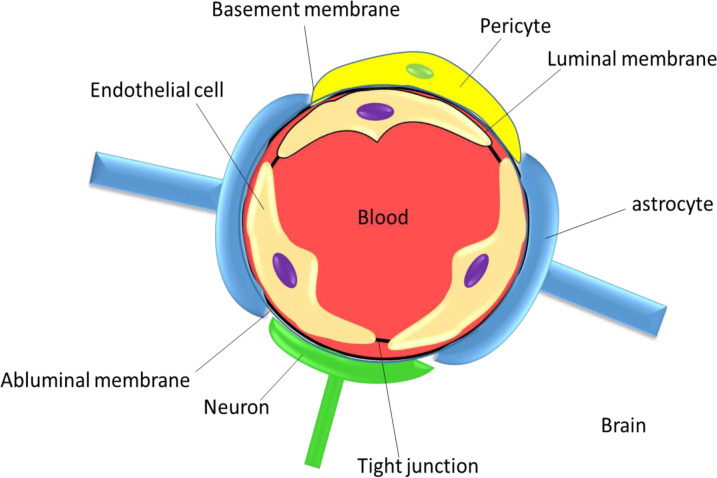
Blood-brain barrier anatomy

The central nervous system (CNS) has developed a variety of barriers to defend itself from circulating blood cells, neurotoxic chemicals, and pathogen invasion (Kadry et al., 2020[47]). These varying degrees of permeable structures include the blood-brain barrier (BBB), blood-cerebrospinal fluid (CSF) barrier, blood-spinal cord barrier, and blood-retinal barrier (Shityakov and Förster, 2018[90]). The BBB is a very effective defense mechanism that utilizes a distinctive structural arrangement mostly observed in blood vessels located in the brain. The BBB consists of a layer of no-fenestrated endothelial cells surrounded by smooth muscle cells, pericytes, and astrocytic projections (Daneman and Prat, 2015[15]). Its main function is to control the movement of ions, chemicals, and cells from the bloodstream into the CNS. The protective barrier plays a crucial role in safeguarding the brain from potential damage under normal circumstances (Blasi et al., 2007[12]; Ding et al., 2014[17]). However, its exceptional selectivity presents a significant obstacle when it comes to delivering drugs for the treatment of different neurological illnesses. Conquering this obstacle is crucial for therapy to be effective (Kadry et al., 2020[47]; Mc Carthy et al., 2015[62]). BBB is a semi-permeable membrane barrier found at the point where blood and cerebral tissue meet (Oller-Salvia et al., 2016[73]). It is made up of pericytes embedded in the capillary basement membrane, tight junction, astrocyte end-feet covering the capillary, and endothelial cells of the capillary wall (Saraiva et al., 2016[85]) (Figure 1(Fig. 1)).

Pericytes: they directly cover around 20 % of endothelial cells. They appear to control brain capillary blood flow by contracting and relaxing in response to a variety of vasoactive stimuli. Astrocytes: the endothelial cells of the BBB are surrounded by astrocyte cell protrusions that biochemically support these cells. Basal membrane: which is primarily formed of laminin, collagen type IV, proteoglycans, heparan sulphate, fibronectin, and other extracellular matrix proteins, is where endothelial cells and pericytes are embedded. In pathological states, the breakdown of this extracellular matrix is directly related to increased BBB permeability (Kadry et al., 2020[47]; Li et al., 2015[55]; Shityakov and Förster, 2018[90]). The BBB results from the selective tight junctions between the endothelial cells, which limit solute movement. These tight junctions, which are made of smaller subunits of transmembrane proteins continually attach endothelial cells at the interface between blood and the brain and prevent the paracellular permeability (Ding and Ma, 2015[18]; Saraiva et al., 2016[85]). 

There are two ways for molecules to cross the BBB:

### Paracellular pathway:

Transport of chemicals across an epithelium through the intercellular gaps between epithelial cells is known as paracellular transport, which is a passive transport mechanism. Tight junctions are essential for the paracellular movement of macromolecules across the epithelium, according to a substantial body of research. For particles to penetrate through the paracellular route, it is the main rate-limiting channel. The tight junctions consist of a complex mixture of intracellular plaque proteins, several regulatory proteins that attach the transmembrane proteins to the actin cytoskeleton, and transmembrane integral proteins such as claudins, occludin, and junctional adhesion molecules (Shamloo et al., 2016[87]).

### Transcellular pathway:

Uses a variety of mechanisms, including passive diffusion, transcytosis, and receptor-mediated transport. The physical and chemical properties that affect BBB permeability include molecular weight (only the molecules under 400-600 Da), charge (Positive Charge), lipid solubility, surface activity, and relative molecule size. While the entry of hydrophilic molecules is typically suppressed, the enormous surface area of the endothelium membrane provides an efficient transcellular passive diffusion channel for lipid-soluble drugs and tiny gaseous molecules (Kadry et al., 2020[47]; Shamloo et al., 2016[87]).

As mentioned above, these properties of the BBB prevent the pharmacological therapy of some neurological disorders from reaching the brain. It should be noted that the existence of the P-glycoprotein pump in the BBB, which enables the recognition of molecules necessary for the brain to enter the brain and the expulsion of other molecules, which includes medicines, represents a further barrier for drugs crossing the cerebral capillary endothelium and entering the brain parenchyma. For these reasons, various strategies have been investigated in order to improve the permeation of drugs across the BBB (Jiang et al., 2016[45]; Miao et al., 2019[65]).

### Approaches or strategies to overcome BBB

### Invasive technique 

a) Blood-brain barrier transient disruption

This method makes use of unpleasant substances or hyperosmotic solutions to decrease the brain's endothelial cells by dissolving tight junctions, allowing various chemicals to enter the cerebral tissue. However, it is important to keep in mind that this particular approach does include some disadvantages, it may damage the BBB's integrity and physiological activities, potentially causing the build-up of neurotoxic, xenobiotic, and exogenous substances, which could harm the CNS (Bang et al., 2017[7]; Gao et al., 2017[29]).

b) Intracerebroventricular and intrathecal infusion

The techniques used in this method include the administration of therapeutic proteins by intraventricular infusion, which involves the direct injection or infusion of these proteins into the cerebrospinal fluid (Dong, 2018[21]).

### Non-invasive technique

The majority of non-invasive approaches include pharmacological tactics that can change the way medications are transported over the BBB like increasing lipid solubility, using transport or carrier systems, inhibition of efflux transporters, trojan horse approach, chimeric peptide, monoclonal antibody fusion proteins, gene therapy, intranasal drug delivery and nanoparticle technologies (Sharma et al., 2021[88]; Tam et al., 2016[96]). This strategy focuses mostly on using nanotechnology to release drugs into the brain. The current efforts are mostly concentrated on improving NPs' capacity to accurately target the therapeutic site, hence reducing the doses of medicines released at unwanted places (Sharma et al., 2021[88]; Yamamoto, 2018[110]).

## Nanoparticles

Nanotechnology has been advancing rapidly over the last 20 years and is now employed extensively in diagnosis, treatment of disease and to enhance the therapeutic transport across the BBB. One of the most promising of these platforms for enhancing the results of existing brain therapeutic delivery is the creation of nanosystems, particularly "nanoparticles" (NPs) (Jelinek, 2015[43]). Nanoparticles are material particles with a dimension of between 1 and 100 nanometers (nm), this term is occasionally applied to particles larger than 500 nm, the particles smaller than 1 nm typically referred to as atom clusters. In 1995, the first successful delivery of a medication across the BBB took place. Hexapeptide dalargin, an antinociceptive peptide that is unable to penetrate the BBB on its own (de Mello Donegá, 2014[16]; Jelinek, 2015[43]).

It was delivered intravenously while covered with polysorbate 80-coated nanoparticles. This was a significant development in the field of nanoparticle drug delivery, and it aided in the advancement of study and development for nanoparticle delivery system clinical trials (de Mello Donegá, 2014[16]). Since NPs are not simple molecules in and of themselves, they are made up of three layers: **(1)** The surface layer, which can be functionalized with a wide range of small molecules, metal ions, surfactants, and polymers. **(2)** The shell layer, which is chemically distinct from the core in every way. **(3)** The core, which is effectively the center of the NP and typically refers to the NP itself. NPs can have negative, Zwitter ionic, or positive charges. Negatively charged spheres are frequently employed in intravenous applications (Tripathy et al., 2023[100]).

Nanoparticles can exhibit different dimensionalities, namely zero-dimensional, one-dimensional, two-dimensional, or three-dimensional structures. Zero-dimensional nanoparticles, such as nanodots, possess fixed dimensions at a single point in space. One-dimensional nanoparticles, exemplified by graphene, are characterized by only one parameter defining their structure. Two-dimensional nanoparticles, for example, carbon nanotubes, possess dimensions in two directions while maintaining a negligible thickness. Lastly, three-dimensional nanoparticles, such as gold nanoparticles, exhibit dimensions in all three dimensions. The morphology, dimensions, and structure of nanoparticles exhibit considerable variety, including irregular or spherical shapes, as well as cylindrical, conical, tubular, spiral, hollow core, and flat configurations (Gautam et al., 2022[30]). The surface may exhibit irregularities and possess either a uniform or asymmetrical configuration.

NPs can be categorized as either natural when substances like proteins (albumin), polysaccharides, chitosan, and others are used. But the extremely popular polymers polylactic-co-glycolic acid, polyethyleneimine, polyesters, or inorganic materials like gold, silica, or alumina can be used to create synthetic NPs (de Mello Donegá, 2014[16]; Jelinek, 2015[43]). NPs can entrap, adsorb, or covalently bind medications to deliver them into cells. The capability of functionalization with various ligand types is another crucial aspect of NPs. The four main categories of ligands are the followings: **(1)** those that can mediate protein adsorption such as polysorbate80 (P-80), **(2)** those that can interact directly with the BBB such as transferrin proteins, antibodies, or peptides, **(3)** those that can increase hydrophobicity such as amphiphilic peptides and **(4)** those that can enhance blood circulation such as polyethylene glycol (PEG) (Gautam et al., 2022[30]).

NPs have several advantages over conventional therapeutic dosage forms, including **(1)** the ability to carry medications and direct them to affected areas, **(2)** encapsulate one or more pharmaceuticals, and **(3)** control the drug release. Encapsulation stops drug release before it reaches the target tissues, which is important for toxicity problems typically connected to non-specific drug distribution in target and off-target tissues. However, NCs are particularly well suited for bypassing the BBB and successfully delivering medicines to the brain through established endogenous BBB transport routes. Therefore, in the sciences of medicine and biology, NPs are receiving more attention (Gautam et al., 2022[30]; Joshi and Joshi, 2022[46]).

### Classification of nanoparticles

**Figure 2:**
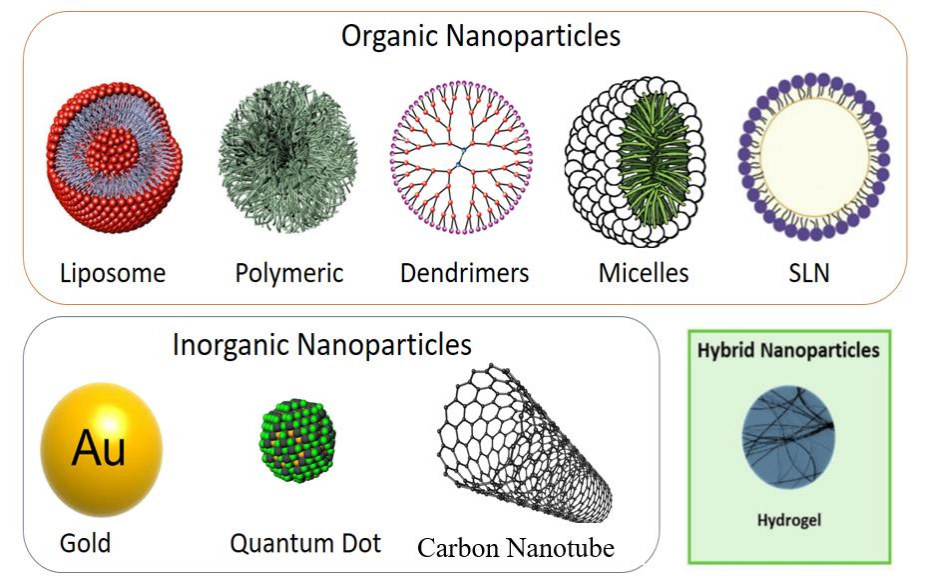
List of different types of nanoparticles with their properties

There are numerous types of nanoparticle (NP)-based medication delivery systems that have been developed. NPs can be broadly categorized into organic and inorganic nanoparticles (ONPs and INPs) based on their composition. ONPs are composed of carbon-based nanomaterials, while INPs do not contain carbon and are composed of inorganic elements e.g., silicon, gold and silver (Kim et al., 2020[51]; Nikalje, 2015[71]). However, there are some NPs composed of allotropes of carbon e.g., fullerenes, carbon nanotubes, graphene-based nano systems or graphene quantum dots (Figure 2(Fig. 2)).

Regarding size and shape control, convenience of preparation, and functionalization, INPs offer benefits over ONPs. Inorganic nanomaterials exhibit interesting mechanical, optical, physical and electrical phenomena at the nanoscale, which can be tailored through changes in material, phase, shape, size and surface characteristics. However, INPs also have disadvantages. INPs frequently require the addition of a biocompatible surface in order to prevent toxicity, especially for heavy metals (Barani et al., 2021[8]; Bastogne, 2017[9]; Kim et al., 2020[51]; Nikalje, 2015[71]). INPs may not degrade, which raises questions about their biocompatibility and biosafety. On the other hand, because carbon makes up a significant portion of our bodies, ONPs tend to have good biocompatibility. However, one of their drawbacks is that they are more difficult to prepare (Upadhyay, 2014[102]). Therefore, if we decided to categorize the various NP functions, we could say that ONPs are more interesting for drug delivery due to their higher biocompatibility and safety, while INPs are better for functionalization and monitoring purposes or for alternative treatments like photothermal therapy and hyperthermia. In order to integrate INPs and ONPs' benefits into a single carrier, hybrid Nps frequently blend the two (Khan et al., 2018[49]; Satapathy et al., 2021[86]) (Table 1(Tab. 1)).

### Types of nanoparticles

### Organic nanomaterials

**Polymeric nanoparticles: **Nanoparticles are solid colloidal particles with sizes typically between 60 and 200 nm. They have a polymeric core matrix in which pharmaceuticals can be embedded and are well suited for drug delivery due to their regulated drug release and targeting effectiveness. They are being extensively employed for the development of drug delivery carriers that can penetrate the blood-brain barrier (Abadeer and Murphy, 2016[1]; Nikalje, 2015[71]). Additionally, they can prevent the reticuloendothelial system from phagocytosing them, which will increase the concentration of medicines in the brain. For the treatment of AD, *in vitro *research demonstrated that the drug delivery to the brain could be increased by utilizing of polymeric nanoparticles, which causes a reduction in oxidative stress, inflammation, and plaque accumulation. Furthermore, an efficient target-specific delivery for application in brain cancer therapy was produced by the *in vivo *investigation involving the simultaneous delivery of the anti-cancer drug cisplatin and the antioxidant agent boldine utilizing polylactide-co-glycolic nanocarriers. Through the invention of a penetrating amphiphilic polymer-lipid nanoparticles' system, docetaxel can now be delivered for the treatment of brain metastases. The *in vivo* experiments showed that the nanoparticles accumulated at the tumor site and successfully suppressed tumor development, increasing median survival in comparison to an equivalent dose of clinically utilized docetaxel fluid formulation (Khan et al., 2018[49]; Nikalje, 2015[71]; Elzoghby et al., 2016[23]; Posadas et al., 2016[80]).

**Liposomes: **A lipid bilayer encircles a hydrophilic core in liposomes. Liposomes' amphiphilic properties allow for the encapsulation of a wide range of medicines with various polarity. Because lipids degrade rapidly in the digestive tract, it is the best to provide liposomes to the brain via (IV) or (IN) routes. Due to their ability to pass the blood-brain barrier and deliver the right amount of medications to the brain, liposome applications are typically used to treat brain tumors (Nikalje, 2015[71]; Raman et al., 2020[82]). NPs should never be larger than 200 nm because it is thought that some pores in human brain tissue are larger than 200 nm, although the majority are slightly larger than 100 nm. However, because liposomes are soft-matter NPs and hence highly flexible, they have advantages in this situation since they can pass through pores even if their sizes are greater than pore diameters (Mistretta et al., 2023[66]; Nikalje, 2015[71]). The disadvantage of such a soft composition is that liposomes are more fragile than, for example, other ONCs such as polymeric nanoparticles. According to numerous studying liposomal formulations have been used to deliver anti-cancer medications such as methotrexate, 5-fluorouracil, paclitaxel, doxorubicin, and erlotinib (Nikalje, 2015[71]).

**Dendrimers: **Dendrimers are globular, hyperbranched polymeric structures that resemble trees in structure. Dendrimers typically consist of a central core (a single atom or group of atoms), generations, repeat structural units connected to the core, and surface functional groups that may have positive, negative, or neutral charges. Dendrimers provide a number of benefits for drug delivery to the brain (Miao et al., 2019[65]; Shamloo et al., 2016[87]). First, their synthesis is precisely controlled, and a wide variety of ligands can be functionalized on their surface to promote BBB crossing. Then, because of their hyperbranched structure, these ligands are very well covered on the dendrimer surface, improving cellular recognition. Additionally, their small size and monodisperse composition enable better regulation of brain uptake. Moreover, the possibility of conjugating their cationic surface groups with nucleic acids makes dendrimers potential ONCs for gene therapy (Nikalje, 2015[71]; Thomsen et al., 2015[99]). Dendrimers have been used in the treatment of brain cancer, stroke, neuroinflammation, and neurodegenerative illnesses. AD has been treated with the anti-epileptic medication carbamazepine encapsulated (Nikalje, 2015[71]).

**Micelles: **Micelles are amphiphilic molecules with a particle size of 5 to 50 nm. Under certain concentration and temperature parameters for the aqueous solution, micelles spontaneously form. Micelles have gained attention for their use in the delivery of poorly water-soluble compounds, their capacity to provide sustained and regulated release, their ability to keep pharmaceuticals chemically and physically stable, and their improvement of therapeutic bioavailability (Baig et al., 2021[4]; Nikalje, 2015[71]). 

**Solid lipid nanoparticles: **Particles with a solid lipid core that exist at body and room temperatures are known as solid lipid nanoparticles (SLNs). As a result of their lipophilic characteristics, SLNs can be employed as drug carriers by passing across the BBB and into the CNS via an endocytosis mechanism. By coating and conjugating SLNs, it is possible to reduce the cytotoxicity of SLNs and enhance the amount of medication delivered to the brain (Abadeer and Murphy, 2016[1]; Nikalje, 2015[71]).

### Inorganic nanoparticles

**Gold Nanoparticles: **Gold Nanoparticles are inorganic particles having a gold core and surface ligands bound by covalent or non-covalent bonds. The shape (spheres, shells, or rods) and size of the particles can be altered depending on the synthesis process. Low concentrations of AuNPs of 10-15 nm were able to penetrate the BBB and reach the brain in a number of *in vivo* investigations on animals. However, the liver and blood contained the vast bulk of the injected amount. Additionally, the amount of gold discovered in the brain varied depending on the AuNP dose, with no evidence of saturation at the studied doses. This implies that the AuNPs might pass across the BBB *via* a non-saturable channel like passive transmembrane diffusion or the paracellular pathway (Bastogne, 2017[9]; Nikalje, 2015[71]; Posadas et al., 2016[80]).

Both the treatment of Parkinson's disease with L-DOPA functionalized multi-branched nanoflower-like gold nanoparticles and the treatment of AD with gold nanoparticles functionalized with -amyloid-specific peptides have been studied, and both have demonstrated improved blood-brain barrier permeability across *in vitro* models. It has also been investigated how the size of gold nanoparticles coated with insulin affects their ability to cross the blood-brain barrier. The findings demonstrated that the 20 nm-diameter nanoparticles had the widest biodistribution and accumulation in the brain (Nikalje, 2015[71]; Posadas et al., 2016[80]).

**Carbon Nanotubes: **A category of nanomaterials known as carbon nanotubes is composed of tubes made of graphite sheets with nanoscale diameters. Carbon nanotubes can have one or more walls, open ends, or fullerene caps to seal them. Drug delivery for the treatment of brain cancer has been used with polymer-coated carbon nanodots and chemically functionalized multi-walled carbon nanotubes. Both *in vitro* and *in vivo* studies revealed that the blood-brain barrier was penetrated and that tumors had increased absorption (Baig et al., 2021[4]; Nikalje, 2015[71]).

**Quantum Dots: **In terms of structure, QDs have a metalloid crystalline core, which will eventually determine the type of light it emits according on its composition and size. Cadmium and tellurium, which both form the core of QDs and have an average diameter of 2 to 10 nm (200-10,000 atoms), are one of the primary precursors utilized in the assembly of these quantum dots in nanotechnology (Bastogne, 2017[9]; Nikalje, 2015[71]). Among the known quantum dots are those made of carbon, selenium, silver, silicon and gold. Graphene quantum dots (GQDs) are currently receiving attention. One of the most recent additions to the group of carbon-based nanomaterials is GQDs. They are currently said to be helpful in treating Alzheimer's and Parkinson's disease. Additionally, it is now known that GQDs have antimicrobial and anti-diabetic properties. They are also currently being extensively tested for use in medicine delivery. They have good potential for medication delivery across the blood-brain barrier. GQD can also be used to deliver medication specifically to tumors (Abadeer and Murphy, 2016[1]; Nikalje, 2015[71]; Posadas et al., 2016[80]).

### Nanoparticles' synthesis

**Figure 3:**
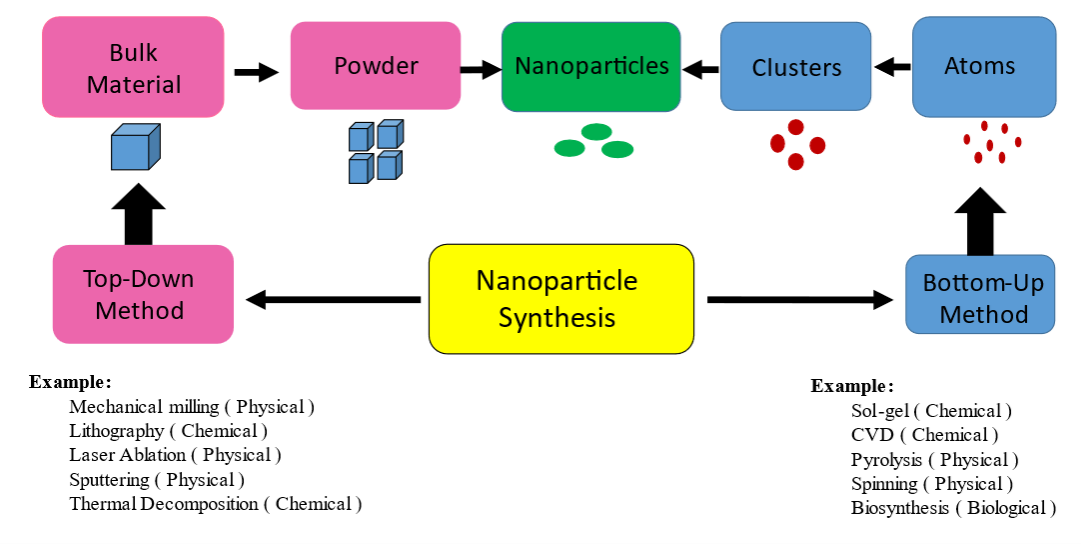
Schematic representation illustrating the various approaches used for synthesis of nanoparticles

**Figure 4:**
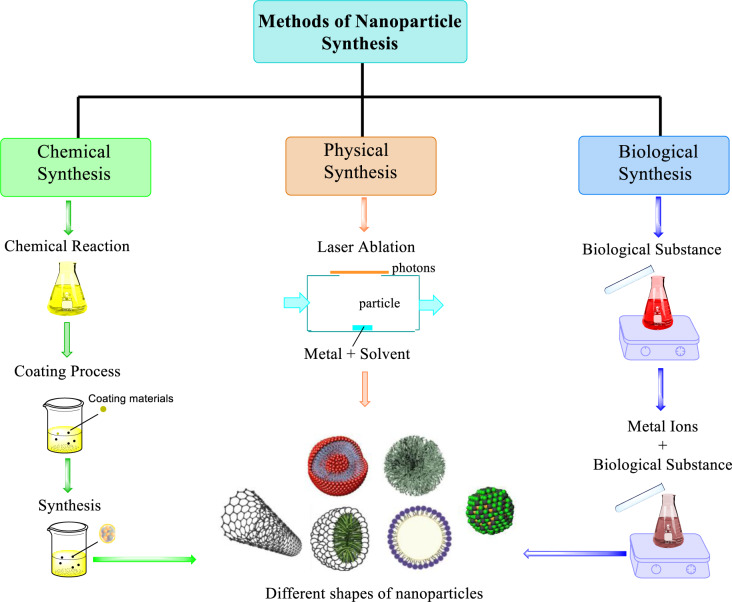
Conventional methods of synthesis of nanoparticles

Synthesis of nanoparticles is a term used to describe production processes, top-down and bottom-up are the two most common techniques. Top-down method refers to the process of breaking down large materials into smaller ones. The bottom-up method depends on forming nanoparticles from atomic-sized components (Figures 3(Fig. 3) and 4(Fig. 4)). In general, nanoparticle synthesis can be carried out *via* physical, chemical, and biological methods, while the precise synthesis method depends on the material being created (Baig et al., 2021[4]; Jeyaraj et al., 2019[44]).

### Top-down method:

**Mechanical milling:** Mechanical milling is a physical process that transfers kinetic energy from the grind medium to the substance that is being reduced. Consolidation and compaction is an industrial-scale procedure that involves milling several components in an inert atmosphere, are used to create material with improved characteristics (do Carmo Lima Carvalho et al., 2023[20]).

**Lithography:** The method of lithography involves printing a necessary shape or structure on a light-sensitive substance whereas only removing a small amount of the material to produce the desired shape and structure (do Carmo Lima Carvalho et al., 2023[20]). The ability to manufacture anything from a single nanoparticle to a cluster with the desired shape and size is one of nanolithography's key advantages, but the main disadvantage is the need for advanced and expensive equipment (Khandel et al., 2018[50]).

**Laser Ablation:** Laser ablation synthesis produces nanoparticles by striking the target material with a strong laser beam. Due to the high energy of the laser irradiation used in the laser ablation process, the source material or precursor vaporizes, resulting in the creation of nanoparticles without the need for any chemical or stabilizing agents (Ealia and Saravanakumar, 2017[22]; do Carmo Lima Carvalho et al., 2023[20]).

**Sputtering:** Sputtering is a technique for creating nanomaterials by hitting solid surfaces with high-energy particles like plasma or gas, depending on the energy of the incident gaseous ions, physically eject tiny atom clusters. Sputtering typically involves depositing a thin coating of nanoparticles, followed by annealing (Ijaz et al., 2020[41]).

**Thermal decomposition:** Thermal decomposition is an endothermic chemical breakdown of a substance caused by heat, which destroys its chemical bonds. The temperature at which an element begins to chemically break down is known as the decomposition temperature. The metal is broken down at particular temperatures in a chemical reaction that yields secondary compounds, creating the nanoparticles (Thomsen et al., 2015[99]).

### Bottom-up method:

**Sol-gel:** The sol is a colloidal suspension of particles in a liquid phase. The gel is a solid macromolecule suspended in a liquid. Due to its simplicity, the majority of nanoparticles can be produced with this method, sol-gel is the most used bottom-up approach. It is a wet-chemical method that uses a chemical solution as a precursor for an integrated system of discrete particles. Precursors for the sol-gel method are frequently metal oxides and chlorides. The precursor is then mixed with the host liquid by sonication, shaking, or stirring, creating a system with a liquid and solid phase. Using a variety of techniques, including centrifugation, filtering, and sedimentation, a phase separation is performed to recover the nanoparticles, and the moisture is then further removed by drying (do Carmo Lima Carvalho et al., 2023[20]; Ijaz et al., 2020[41]).

**Chemical Vapor Deposition (CVD):** The process of depositing a thin layer of gaseous reactants onto a substrate is known as chemical vapor deposition. The deposition is carried out at room temperature by mixing gas molecules, in a reaction chamber. A heated substrate that comes into touch with the mixed gas experiences a chemical reaction (Bastogne, 2017[9]). A thin film of product is created during this reaction and is recovered to be used. The influencer of CVD is substrate temperature (Ealia and Saravanakumar, 2017[22]). 

**Pyrolysis:** The most widely utilized method for producing nanoparticles on a big scale is pyrolysis. It involves using flame to burn a precursor. The precursor is either a liquid or a vapor that is fed under high pressure through a small hole into the furnace, where it burns. The nanoparticles are then air-classified from the combustion or by-product gases. To get a high temperature for simple evaporation, use plasma rather than a flame (Ijaz et al., 2020[41]; Khandel et al., 2018[50]). 

**Spinning:** A spinning disc reactor creates nanoparticles by spinning them together (SDR). The physical parameters, such as temperature, can be adjusted by rotating a disc inside a chamber or reactor. To remove oxygen and prevent chemical reactions, the reactor is often filled with nitrogen or other inert gases (Khandel et al., 2018[50]). The liquid, such as water and precursor, is poured into the disc as it is rotating at various speeds. The atoms or molecules are fused together by the spinning, and the result is precipitated, collected, and dried.

**Biosynthesis:** Biosynthesis sometimes known as "green synthesis," is an eco-friendly method for creating non-toxic, biodegradable nanoparticles (Pinheiro et al., 2021[78]). Instead of using conventional chemicals for bio-reduction, biosynthesis uses bacteria, plant extracts, fungi, and yeast combined with the precursors to make nanoparticles (Jeyaraj et al., 2019[44]).

### Methods of nanoparticles synthesis:

**Physical method:** Physical method uses mechanical pressure, high-energy radiation, thermal energy, or electrical energy to abrade, melt, vaporize, or condense the material to produce NPs. It doesn't need any chemical material but has a high waste product and less productivity with many difficulties in size and shape tenability that's why it's not a good method for preparing NPs with desired shape and size and has less stability. The physical methods of synthesis include (mechanical milling, sputtering, laser ablation, flame pyrolysis, spinning) (Navarro and Salas, 2022[70]).

**Chemical method:** Accomplished *via* chemical reduction using several organic and inorganic reducing agents, electrochemical methods, physicochemical reduction. The advantages of this method include high yield, controlled size, stability, easy preparation, and cost efficiency. Most common techniques in this field are the following: Chemical vapor deposition, the sol-gel method, lithography, thermal decomposition (Khandel et al., 2018[50]; Navarro and Salas, 2022[70]).

**Biological method:** The biological process involves creating nanoparticles utilizing plant extract (leaf, stem, root and fruit), bacteria, fungi, and microorganisms and there are many advantages of this method like: protecting the human health, low cost, economical, lesser waste product, and fewer accident. And it is the best method due to effectiveness, simplicity and non-toxicity (Kaur et al., 2023[48]).

## Mechanism to Cross the BBB

### Adsorptive-Mediated Transcytosis (AMT) 

The AMT gives nanoparticles a way to cross the BBB and enter the brain. This process is started by an electrostatic interaction between positively charged ligands and the negative-charged cell membrane. The glycocalyx of the BBB's endothelial cells, which is made up of the heparin sulphate proteoglycans (HSPGs) glypican and syndecan, covers their phospholipid-rich membrane (Dong, 2018[21]). On this side of the BBB, sialo glycoproteins and sialo glycolipids have a lot of carboxyl groups. All of them give rise to the luminal side of the BBB's significantly negative charge. As a result, AMT can be facilitated by the electrostatic interactions between the cationic groups of ligands conjugated on the surface of nanoparticles and the negative moieties exposed at the luminal surface of cerebral endothelial cells (Ding et al., 2020[19]).

The mechanism used to explain how NPs interact with the luminal surface of the BBB is based on how well their surfaces have been functionalized. By adding a positive charge to the NPs surface, this interaction can be facilitated given that endothelial cells have negative charges. This problem can be realized using a variety of techniques. The construction of NPs from materials that are positively charged at physiological pH is one option (Bellettato and Scarpa, 2018[10]).

### Receptor-Mediated Transcytosis (RMT)

The RMT is one of the most recently used drug delivery methods using functionalized NPs through the BBB endothelium. In endothelial cells, transcytosis begins with vesicular carriers bringing extracellular cargo within the cell. It is dependent on particular receptors existing on the luminal surface of cells (Masserini, 2013[61]). The justification for using this technique with NPs is that the cellular mechanisms already in place for transporting macromolecular cargoes may work just as well for NPs after they have been functionalized to engage with the same receptors. The cargo is then processed by several intracellular organelle pathways to the appropriate recycling, degradation, or transcytosis to the contralateral side (Lombardo et al., 2020[59]; Teleanu et al., 2018[97]). It is anticipated that by identifying and studying novel receptors that may be employed for receptor-dependent endocytosis, more efficient brain medication delivery systems will be produced because these absorption techniques are largely resistant to lysosomal degradation (Sukanya and Arabinda, 2022[94]; Teleanu et al., 2018[97]).

### Crossing the BBB without functionalization

Despite the fact that practically all nanomaterials belong to the BBB impermeable class, some exceptions have recently been reported. For example, gold and silica NPs have been demonstrated to enter the brain and accumulate in neurons even in the absence of any specific functionalization, with a mechanism that is largely still understood. In the case of silica, the results indicated that NPs administered to rodents via intranasal instillation entered into the brain and especially deposited in the striatum (Liu and Xu, 2022[56]; Mistretta et al., 2023[66]). According to the findings in the silica example, NPs given to animals by intranasal instillation penetrated the brain and were particularly deposited in the striatum. By using X-ray microtomography, confocal laser, and fluorescence microscopy, accurate particle distribution of gold nanoparticles in the brain was examined *ex vivo*. The hippocampus, the thalamus, the hypothalamus and the cerebral cortex were the places where the particles accumulated most frequently, according to the authors (Pardridge, 2016[75]).

### Retrograde Transport

Some types of nanocarriers may be able to move from CNS neuronal cell bodies to peripheral nerve terminals via transsynaptic retrograde transport. NPs treated with PEI and other polyplexes showed active retrograde transport via neurites, but they are unable to mediate effective biological activities once they reach the neuronal body, according to studies in this area (Liu and Xu, 2022[56]; Mohammadi et al., 2017[67]).

### BBB breakdown

Neuroinflammatory disorders cause BBB degradation. The paracellular permeability of NPs can be increased by temporarily and irreversibly opening the tight junctions at the BBB and other locations. Blood-brain barrier disruption therapy, in particular, is a powerful, intensive method of administering medication to brain tumors. As a result, only NPs smaller than roughly 20 nm can use this channel to enter the brain across the BBB since tight junctions can only be opened to a certain extent (Dong, 2018[21]; Liu and Xu, 2022[56]).

### Monocyte/macrophage infiltration

**Figure 5:**
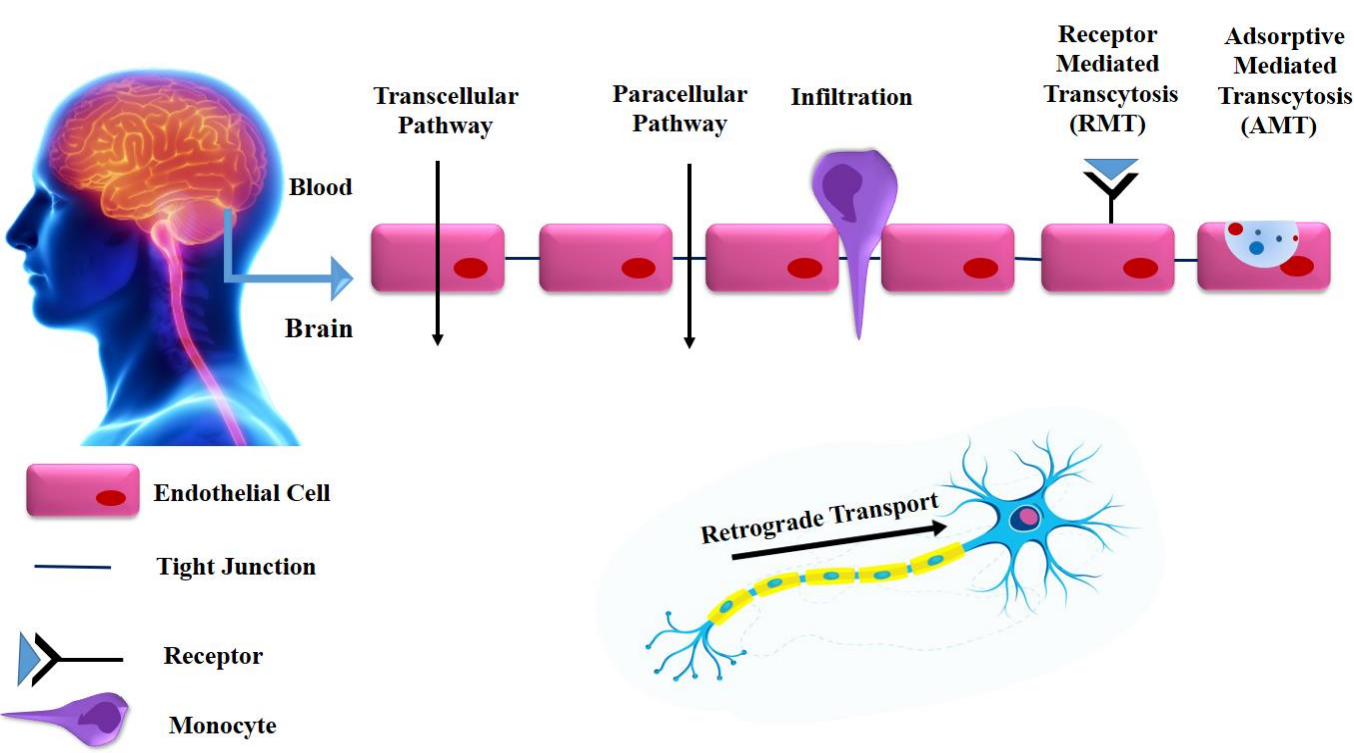
Schematic diagram of the mechanisms for crossing the BBB

This mechanism plays a critical function in the CNS in neuroinflammation, lesion formation, and brain injury in neurological illnesses such multiple sclerosis (MS) and stroke. Because anti-inflammatory medications can target the chemokines that regulate cellular trafficking and cell adhesion molecules, understanding the active periods of cell infiltration during CNS diseases is crucial. BBB crossing by immune-activated macrophages looks to offer potential plans for NP-based therapeutic developments in the future. This tactic could be implemented at least by two different ways: (1) by incorporating NPs into activated monocytes that are being employed as Trojan horses to enter the brain, and (2) by creating NPs that mimic activated monocytes (Paterson and Webster, 2016[77]; Wei et al., 2016[105]) (Figure 5(Fig. 5)).

### Trojan monocytes for brain NP delivery

In order to avoid RES clearance and gain longer circulation times for improved tissue uptake, nanoparticulate drug delivery devices have been utilized. However, it might be argued that increasing NPs phagocytosis by monocytes is an unconventional method of delivering medications packaged on NPs to the brain. Monocytes may act like Trojan horses and carry their cargo into the brain after phagocytosing NPs (Bakhshinejad et al., 2015[6]; Pinto et al., 2022[79]). 

### Monocytes that mimic activated NPs

Drug-loaded NPs systemically delivered for therapeutic purposes must be able to bypass the immune system, overcome bodily biological barriers, and localize in target tissues. It is predictable that research on NPs that mimic immune cells may be effective in treating disorders associated with the brain and will receive a stimulus in this direction in the coming years because several studies show increased passage of monocytes across the BBB in a variety of pathological conditions (Raikwar and Jain, 2022[81]).

## Applications of Nanoparticles

Clinical uses of nanoparticles are crucial for the early diagnosis and therapy monitoring of conditions like cancer and neurological diseases, and they are a vital resource for both clinicians and patients since they improve patient management and survival and quality of life (Mohammed et al., 2023[68]). Since they have a number of benefits, including targeting efficiency, non-invasiveness, biodegradability, stability, and controllability to load and release drugs, nanoparticles are being used more frequently to diagnose brain disorders or to help with drug delivery across the blood-brain barrier (Mohammed et al., 2023[68]; Zhou et al., 2021[114]).

### Brain tumors 

The current standard of care includes a careful surgical resection followed by the oral administration of temozolomide while receiving concurrent radiotherapy, however, this has not been able to significantly slow the progression of the disease (Wang and Wu, 2017[104]). New therapeutic approaches are urgently needed since primary brain tumors in adults are one of the most prevalent and lethal types (Silveira et al., 2023[91]). In recent years, polymeric nanoparticles containing the chemotherapeutic drug paclitaxel have been used by capitalizing on one of the few biological characteristics of this type of tumors that is recognized. Because of this, circulating nanoparticles containing paclitaxel may cross the BBB and build up in the tumor (Iv et al., 2015[42]; Noor et al., 2022[72]). Similar approaches, encapsulating paclitaxel with carbon nanoparticles, have been shown to improve the outcome of head and neck tumors, but again, no data on human volunteers has been demonstrated. A doxorubicin delivery method is currently being tested in human clinical trials in glioblastoma patients (Iv et al., 2015[42]; Ribarič, 2021[83]).

### Alzheimer's Disease (AD)

The main current class of drugs used in AD are acetylcholinesterase inhibitors. Another approach is to block glutamatergic neurotransmission: memantine is a NMDA-receptor antagonist (Liu et al., 2019[57]). There is obviously a need for better therapies because these treatment approaches only offer a modest improvement. However, acetylcholinesterase inhibitor (Rivastigmine) was loaded in SLN using nanotechnology in order to avoid its severe first-pass metabolism and significant side effects following oral administration. Being lipidic in nature, rivastigmine-loaded SLN demonstrated greater drug diffusion than drug solutions containing the crystalline form of the drug (Ostrom et al., 2021[74]; Zhou et al., 2013[113]). Aiming to improve their brain biodistribution, antioxidants were also loaded into SLN as a potential AD treatment. Particularly in neuronal cell lines, astaxanthin-loaded SLN demonstrated a potent neuroprotective effect against oxidative stress (Whittle et al., 2015[106]). Biodegradable polymeric nanoparticles made of polyethylene glycol that have been functionalized with certain antibodies or oligopeptide medications have been used to stop the development of amyloid fibrils, which cause the disease (Yusuf et al., 2021[111]; Zhou et al., 2013[113]).

### Cancer

Metallic nanoparticles have been investigated for their novel biological ability to promote cell death and induce autophagy. Moreover, biological metallic NPs are cytotoxic agents to fight different types of cancer (Hassanzadeh et al., 2017[33]). For the anticancer activity of biological NPs, there are three different mechanisms. The first is the apoptotic pathway, which depends on an elevated quantity of ROS and causes oxidative stress and DNA fragmentation in the malignant cell. Secondly, proteins and DNA interference play a crucial role in the anticancer activity of nanoparticles, as these nanomaterials can disrupt protein functions involved in cancer cell signaling, proliferation, and survival, while also interacting with DNA to induce strand breaks, inhibit replication, and impair repair mechanisms. Thirdly, the interaction of biological nanoparticles with cell membranes alters cell permeability and mitochondrial dysfunction (Bourganis et al., 2018[13]). Liposomes have been used in studies to transport anti-cancer medications including doxorubicin and erlotinib. The findings revealed a much-increased translocation across the blood-brain barrier (Chen, 2019[14]; Hasan et al., 2019[32]; Hussen et al., 2023[39], 2024[37][40]). There is an *in vivo* study, for instance, liposomes and polymeric nanoparticles have been used to co-encapsulate drugs like doxorubicin and paclitaxel, or chemotherapeutics with gene therapy agents. These combination therapies showed enhanced therapeutic outcomes in animal models due to the ability of nanoparticles to target specific tissues or tumors, prolong drug circulation, and co-deliver agents in a controlled manner.

While many of these studies have shown promise *in vivo* models, translating these findings to individualized human therapies is still in its early stages, as challenges like dosing optimization, nanoparticle stability, and long-term safety must be addressed.

### Parkinson's Disease 

Several types of nanoparticles have been explored for Parkinson's disease, which is linked to motor aspects such as rest tremor, bradykinesia, rigidity and postural instability, olfactory dysfunction, cognitive impairment, mental symptoms, and autonomic dysfunction (Bourganis et al., 2018[13]; Mendes et al., 2018[63]). Some of these researches described the use of chitosan nanoparticles for the *ex vivo* delivery of the Parkinson's medications, Pramipexole and Selegiline, two well-known anti-Parkinson drugs (Xiong et al., 2019[109]).

### Stroke

The discovery of methods for both immediate therapy and post-stroke recovery is essential because there are few treatments now available for stroke. Studies on using polymeric nanoparticles, liposomes, metal and metal oxide nanoparticles, as well as inorganic and organic nanoparticles, are currently being done for stroke therapy (Shen et al., 2017[89]; Sun et al., 2016[95]). According to one study, Lexiscan was encapsulated in poly (lactic-co-glycolic acid) nanoparticles functionalized with chlorotoxin as a target ligand that could increase blood-brain barrier permeability. Findings demonstrated increased stroke survival, demonstrating the system's potential for stroke therapy (Hussen, 2023[36]; Sood et al., 2017[93]).

## Current Materials in Nanomedicine

The FDA in the United States and the European Medicines Agency in the European Union have both approved different nanoparticles for use in clinical applications. These nanocarriers contain the desired medications (Fang et al., 2014[26]). Since the first liposome was described in 1965, it took more than three decades for Doxil, the first FDA-approved cancer nanomedicine, to be released in 1995. Following that, the FDA approved further liposomal formulations and albumin-bound nanoparticles for the treatment of cancer and other diseases (Meola et al., 2018[64]; Zhang and Song, 2016[112]).

The approved nanomedicines in treating the brain disease include:

- Doxorubicin, a drug used to treat many different kinds of cancers including breast, bladder, Kaposi's sarcoma, lymphoma, and acute lymphocytic leukemia, but in the field of brain disease it is used to treat glioma (Fan et al., 2014[25]; Hussen et al., 2023[39]; Nam et al., 2018[69]).
- The combination of Borneol and Doxorubicin is used for dual-functional glioma, due to targeting improved area under the curve (AUC) and drug accumulation in brain tumors, prolonged half-life time and enhanced drug accumulation in glioma cells (Gao, 2016[28]; Sood et al., 2017[93]).
- Gold nanoparticles (Au-NPs) and L-DOPA for Parkinson's disease are readily absorbed by brain macrophages and cause no inflammation, effectively permeate the BBB (Kumari et al., 2016[54]).
- Marqibo, used to treat a number of types of cancer, includes acute lymphocytic leukemia, acute myeloid leukemia, Hodgkin's disease, and small cell lung cancer but especially for brain it is used in neuroblastoma (Ezzati Nazhad Dolatabadi and Omidi, 2016[24]; Kuang et al., 2016[53]).
- Onivyde is one of the therapies that is used for brain cancer but it has anticancer activity across a broad range of malignancies, including pancreatic cancer, esophagogastric cancer, and colorectal cancer (Al-Kassas et al., 2017[2]; Lu et al., 2015[60]).
- Invega® Sustenna is used for schizophrenia and schizoaffective disorder (Fu et al., 2018[27]; Teleanu et al., 2019[98]).

## Pharmacokinetics and Side Effects of Nanoparticles

When it comes to delivering pharmaceuticals to the brain, the pharmacokinetics of nanoparticles for drug delivery to the brain is the study of how these tiny carriers interact with the body during absorption, distribution, metabolism, and excretion.

- Absorption: Several methods, including intravenous injection, intranasal administration, and direct injection into the brain, can be used to deliver nanoparticles. The size, surface charge, and surface modification of nanoparticles affect their absorption and can affect their capacity to pass across biological barriers such as the blood-brain barrier (BBB) (He et al., 2024[34]).
- Distribution: After entering the bloodstream, nanoparticles have the ability to move around and disperse all over the body. In order for drugs to be delivered to the brain, nanoparticles need to cross the blood-brain barrier. The size, shape, and surface characteristics of nanoparticles, as well as the BBB's integrity, all affect their distribution (He et al., 2024[34]; Wilhelm et al., 2016[107]).
- Metabolism: The body can metabolize nanoparticles by enzymatic breakdown or reticuloendothelial system (RES) clearance. The metabolic stability and circulation duration of nanoparticles can be affected by changes to their surface characteristics or composition (Blanco et al., 2015[11]; He et al., 2024[34]).
- Excretion: The body can eliminate nanoparticles by a number of processes, including exhalation, hepatic clearance, and renal clearance. Determining the pharmacokinetic profile and possible toxicity of nanoparticles requires an understanding of their excretion mechanisms (He et al., 2024[34]; Tsoi et al., 2016[101]).

Different cell types in different organs and tissues are involved in the body's removal of nanoparticles. The following are some of the main cell types that are involved in the removal of nanoparticles:

- Macrophages: These cells are specialized in absorbing and eliminating foreign particles, including nanoparticles, and are present in tissues all over the body. Significant roles in the clearance of nanoparticles are played by macrophages found in the liver (Kupffer cells), spleen, lungs, and other tissues (Blanco et al., 2015[11]).
- Endothelial cells: These blood vessel-lining cells can interact with nanoparticles and help remove them from the bloodstream. Examples of these cells include sinusoidal endothelial cells found in the liver, spleen, lungs, and other organs.
- Hepatocytes: The liver's metabolic and detoxifying cells have the ability to absorb nanoparticles from the blood and digest them before excreting them from the body in the form of urine or bile (Baimanov et al., 2019[5]; Blanco et al., 2015[11]).
- Renal cells: Via renal excretion, nanoparticles small enough to be filtered by the kidneys can be removed from the bloodstream. Renal cells other than proximal tubular cells are involved in the clearance of nanoparticles.
- Alveolar macrophages: These cells, which are found in the lungs' alveoli, have the ability to collect and eliminate nanoparticles that are inhaled or enter the bloodstream and then make their way into the lungs (Blanco et al., 2015[11]).
- Dendritic cells: These immune response-related antigen-presenting cells can also help remove nanoparticles, especially in regions like lymph nodes that have a high level of immunological activity.
- Splenic macrophages: By filtering blood and ensnaring circulating particles, macrophages in the spleen, such as red pulp macrophages and marginal zone macrophages, aid in the clearance of nanoparticles (Blanco et al., 2015[11]).
- Bone marrow cells: Hematopoietic stem cells and macrophages within the bone marrow can interact with bloodstream-circulating nanoparticles.

Together, these cell types identify, engulf, and digest nanoparticles, ultimately enabling their excretion from the body through a variety of pathways, including renal and hepatic excretion, as well as sequestration in organs like the liver and spleen. In order to minimize potential off-target effects and build successful nanomedicines, it is imperative to comprehend the interactions between nanoparticles and cells. The effective delivery of medications to the brain can be improved while minimizing systemic negative effects by optimizing the design and administration routes of nanoparticles (Baimanov et al., 2019[5]; Blanco et al., 2015[11]).

The fraction of nanoparticles that enter the brain and the other fraction that exit the central nervous system (CNS) might differ according on a number of variables, such as the characteristics of the particles, the method of administration, and physiological circumstances. Usually, peripheral macrophages, endothelial cells, and other cell types outside of the brain remove the bulk of given nanoparticles from the bloodstream, with only a tiny fraction managing to successfully penetrate the blood-brain barrier (BBB) and enter the brain (Park, 2013[76]).

The following are some unwanted effects that may arise from peripheral macrophages, endothelial cells, and other cell types absorbing nanoparticles:

- Off-target actions: When endothelial cells or peripheral macrophages absorb nanoparticles, they may cause inflammation or unanticipated pharmacological actions in these tissues, which might result in off-target effects or systemic toxicity (Blanco et al., 2015[11]; Park, 2013[76]).
- Immunological response: Peripheral macrophage absorption of nanoparticles may initiate immunological reactions, which may result in inflammation or unfavorable immune-mediated outcomes.
- Organ toxicity: When macrophages or endothelial cells absorb nanoparticles into organs like the liver, spleen, or kidneys, it can cause accumulation of these particles in those organs, impairing their functionality and perhaps resulting in negative health repercussions (He et al., 2024[34]; Park, 2013[76]).
- Vascular effects: When nanoparticles interact with blood vessel endothelial cells, they can damage or destroy the integrity of the vascular wall, which can result in thrombotic episodes or vascular toxicity.
- Long-term effects: Prolonged exposure to nanoparticles and peripheral cell absorption may result in cumulative toxicity or the emergence of persistent inflammatory diseases (Blanco et al., 2015[11]).

In general, even though targeted drug delivery to the brain is one of the most promising uses of nanoparticles, their safe and efficient usage in clinical applications depends on our ability to comprehend and mitigate the possibility of off-target effects and systemic toxicity. The goal of ongoing research is to optimize the design and administration techniques of nanoparticles to minimize side effects and maximize their therapeutic advantages (Blanco et al., 2015[11]; Park, 2013[76]).

## Methodology

**Figure 6:**
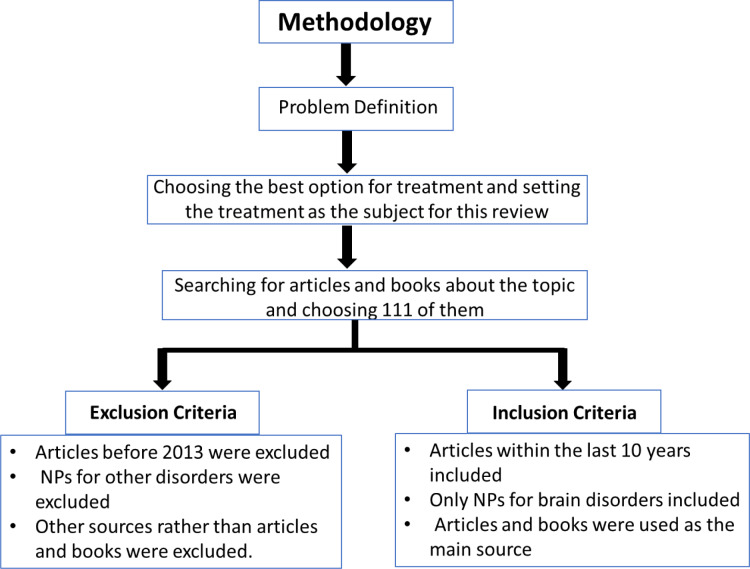
Figure 6 shows the flow chart of research work.

We started to search for an appropriate topic based on finding the best treatment for a serious problem; after a meeting we decided to choose the topic of “Role of nanoparticles for brain drug delivery” because this is a new and an effective technique to reach the CNS especially when there are a lot of brain disorders without treatment; after searching for articles and books on nanoparticles, we decided to use 111 of them, which were closest to our topic (Figure 6(Fig. 6)).

### Inclusion criteria

- Time of publication articles: Most of the articles that we used were within the last 10 years 2013-2023, we tried to choose those articles that were recently done.
- NPs for brain drug delivery: Although NPs are used for treatment of many diseases in different parts of the body but the main and basic goal of this research is specific for those NPs that are used for treating brain disorders.
- The sources of information were only books and articles.

### Exclusion criteria

- Old studies that are done before 2013 are excluded.
- Other applications of nanoparticles for all organs of the body other than brain are excluded.
- The sources like video, interview, data collecting from both experts and people were excluded.

## Conclusion

This review has focused on recent techniques of nanotechnology for delivering medications to the brain because the number of deaths from neurological or neurodegenerative diseases is comparable to that of a world war. The presence of BBB makes it difficult to treat these disorders. This review has demonstrated how the BBB is permeable; how its regulatory mechanisms work; how the academic/pharmaceutical communities are facing significant difficulties in the research and development of innovative drug delivery methods for the treatment of such disorders. A novel and promising strategy is nanotechnology.

Nowadays, a variety of NP types with various characteristics and applications are available for usage in biomedicine. NPs provide clinical benefits for medication delivery, including lower drug dosages, less side effects, longer half-life and the potential to improve drug crossing of the BBB. Although highly promising, the improvement in brain delivery achieved with drug-loaded NPs is still quantitatively restricted compared to free drugs. For a better understanding of the controlling mechanisms, based on various NPs-mediated transport of the medications to the brain, additional research is required. Nonetheless, the significant financial inputs required to enable the translation of preclinical research into actual clinical applications is worthwhile strategic approach.

## Conflict of interest

The authors declare no conflict of interest.

## Figures and Tables

**Table 1:**
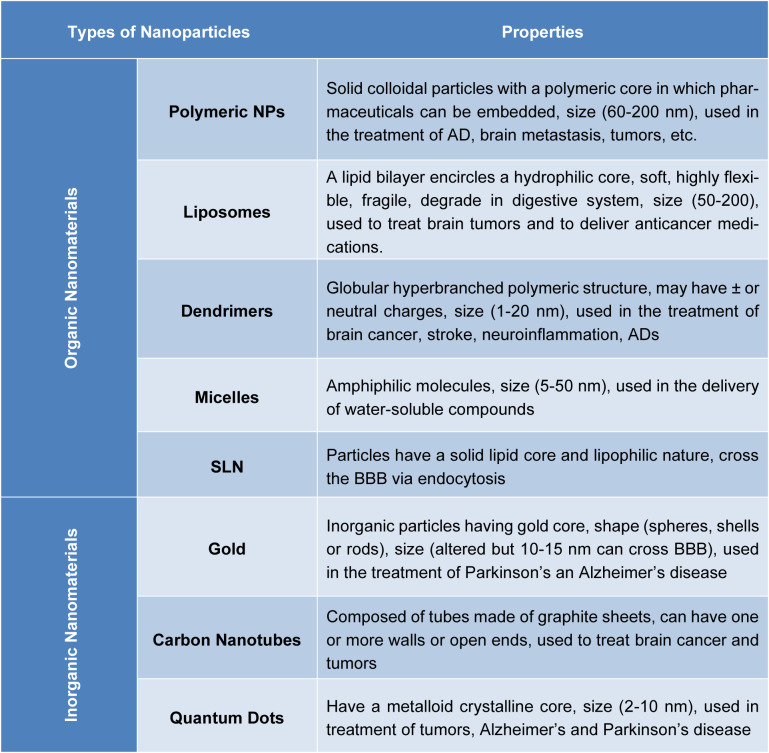
List of different types of nanoparticles with their properties
